# The experience of couples being given an oxygen concentrator to use at home: A longitudinal interpretative phenomenological analysis

**DOI:** 10.1177/1359105315615932

**Published:** 2015-11-29

**Authors:** Ross Thomson, Jennifer L Martin, Sarah Sharples

**Affiliations:** University of Nottingham, UK

**Keywords:** experience, interpretative phenomenological analysis, older person, respiratory problems, treatment

## Abstract

This longitudinal study explores the lived experience of four couples where one person from each couple is prescribed an oxygen concentrator to use at home. Transcripts were analysed using interpretative phenomenological analysis. The findings reported here focus on two super-ordinate themes: ‘the journey of acceptance’ and ‘negotiating changing relationships’. Participants described a gradual process of accepting the device into their lives, the impact on couple’s relationships and the role that expectations have in mediating that process. These themes suggest that patient education that considers the psychological and social issues may prove useful in facilitating the acceptance process.

## Introduction

Chronic obstructive pulmonary disease (COPD), which is the umbrella term for a collection of lung diseases including emphysema and chronic bronchitis, is a global health problem of increasing mortality and morbidity. Symptoms include shortness of breath, reduced exercise tolerance, regular sputum production and wheeze, which have an adverse effect on the ability to carry out daily activities and quality of life (Chapman et al., 2006).

While research on COPD has predominately focused on the patient, a small number of studies have investigated the impact this disease has on close family members. Bergs (2002) described the experience of women caring for husbands with COPD. The effect of COPD on the wife-caregivers’ experience of quality of life was profound. Themes that emerged from the interviews included not having time to worry about their own health, a weakening of marital relationship, living in an emotional straitjacket, walking the road with him to the very end and the prospect of adjusting to a single life. Gabriel et al. (2014) described similar difficulties faced by COPD families related to tensions in relationships, social isolation, sense of powerlessness, emotional strain and uncertainty towards the future. The difficulties and resultant strain on family members reflect the extra responsibilities and new roles undertaken by them as patient illness progresses (Seamark et al., 2004)

Long-term oxygen therapy (LTOT) is established as the only therapy proven to alter the course of later stage COPD by improving survival rates in patients with severe COPD who use oxygen for at least 15 hours a day (Nocturnal Oxygen Therapy Trial Group, 1980). The most common and cost-effective way to deliver oxygen for LTOT patients is by using an oxygen concentrator. Oxygen concentrators run off mains electricity and work by forcing room air through a series of filters, which remove nitrogen and other low-concentration gases, thus concentrating the oxygen levels in the resulting product. This is then delivered to the patient via plastic tubing to either a face mask or nasal cannula (Gibbons et al., 2002).

In addition to improved survival rates, research has also indicated the LTOT may be significantly associated with improvements in health-related quality of life (Eaton et al., 2004), reduced hospital admissions (Ringbaek et al., 2002) and improvements in mood and attitudes (Borak et al., 1996).

Despite these positive outcomes, Ring and Danielson (1997) reported conflicted feelings expressed by patients’ experience of LTOT. The restrictions that the therapy imposes have to be positioned against the advantages the oxygen has on the body. This means that patients go on to describe how they have to put up with and to tolerate LTOT in order to survive.

More recently, research has started to consider the role of family members in relation to LTOT. Kanervisto et al. (2007) compared family dynamics in families of COPD patients without oxygen therapy and in families of COPD patients with LTOT. They described family dynamics in families with LTOT were notably worse in the dimensions of communication (i.e. distorted communications and unclear perceptions) and roles (including role reciprocity and role conflict), but better in terms of individuation, mutuality, flexibility and stability. The dysfunction in the roles of roles and communication in the families with LTOT seems to support some of the experiences of care-giving wives reported by Bergs (2002) in that dysfunctional dynamics in families with severe COPD may weaken the ability of those families to manage in everyday life.

Goldbart et al. (2013) conducted a qualitative study using data from focus groups and interviews with patients who were prescribed LTOT, their informal carers and healthcare professionals. Patients and informal carers identified positive aspects to their treatment in terms of the social benefits of being able to leave the house more and feeling better able to manage their daily lives. This however was offset by the stigma associated with the equipment and the perceived dependency on the therapy.

LTOT is not the only treatment used in the care of patients with COPD. Gale et al. (2015) have discussed patients’ and carers’ experiences with regards to home non-invasive ventilation. This work has highlighted the need to recognise social, technical and experiential issues in adjusting to this particular therapy which bares many similarities with the LTOT literature.

Although the literature has built up a complex picture of the experience of people with COPD on LTOT, the central role the concentrator itself plays in this therapy and its effect on the lives of both patients and partners have not been explored in great detail. While participants in previous studies have been established on oxygen therapy for some time, the aim of this study was to follow couples and describe their experience as they began LTOT to understand the issues associated with the device and how these may alter over time. In order to do this, the study adopted a longitudinal design and selected interpretative phenomenological analysis (IPA) as the analytical approach.

## Method

### Participants

Following approval by the National Health Service (NHS) Ethics Committee (reference: 12/EM/0388), four couples were recruited from an oxygen assessment clinic in the East Midlands of the United Kingdom. The inclusion criterion was that one person in each couple had been assessed by the clinic as requiring an oxygen concentrator to be used in the home as a result of their COPD.

The concentrators encountered in this study were typical of the ones provided by the NHS in the United Kingdom. They were grey in colour, approximately 70 cm tall (about the size of a bedside cabinet) and generated noise levels similar to that of a dishwasher. They weighed 25 kg and had castors, although in reality, the participants in this study did not need to move the device around as they were provided with enough plastic tubing to facilitate movement throughout the house both upstairs and downstairs.

Participant profile characteristics and interview timetable are shown in Table 1.

**Table 1.** Participant characteristics and interview timetable.

	Pseudonym	Age	Interview 1 (Tl)	Interview 2 (T2)	Interview 3 (T3)
Couple 1	Ray (device user)	75	3 days post device delivery	4 weeks after first interview	7 weeks after second interview Device had been removed from home
	Rita (partner)	69			
Couple 2	Sally (device user)	65	6 weeks post device delivery	4 weeks after first interview	4 months after second interview
	Stan (partner)	65			
Couple 3	Tracy (device user)	84	1 day prior to device delivery	4 weeks after first interview (told to stop using device after 2 weeks)	No third interview due to device removal
	Terry (partner)	82			
Couple 4	Wilma (device user)	69	6 weeks post device delivery	4 weeks after first interview	4 months after second interview
	Wally (partner)	69			

### Procedure

All the interviews took place in participants’ homes, and device users and their partners were interviewed together. The first interview was conducted within 6 weeks of receiving the oxygen concentrator. The intention was to interview the couples prior to receiving the device; however, because the time taken from the decision to prescribe the device and the device being delivered into the home was 3 working days, this left only a small window for a mutually convenient time for the interview to take place. The second interview was planned to take place a month after that, and the final interview was planned for 4 months after that. Two of the couples had their device removed during the course of the study, and as a result, post removal interviews were conducted. Consent forms were completed prior to each interview to ensure continued understanding of the process and that participants were willing to continue.

The semi-structured interviews were guided by an interview schedule consisting of 12 open-ended questions. Some of the questions were broad and exploratory in nature and were followed up with more focused prompts when required. Interviews lasted approximately 60 minutes. The interviews were recorded digitally and were subsequently transcribed verbatim.

### Analysis

Transcripts of the interviews were analysed using IPA. This was deemed an appropriate analytic approach as it allows for an in-depth examination of how people make sense of life experiences (Smith and Osborn, 2003). Here, it offered a way of exploring not just the lived experience of being given an oxygen concentrator but also how couples made sense of this over time. IPA does not prescribe a single method of analysing data but suggests a set of common processes which can be applied flexibly while maintaining the analytic focus on the exploration of participants’ experience that is the essence of IPA. This study based its approach to analysis on the six steps suggested by Smith et al. (2009). The analysis differed in that not only were themes looked for across cases but also how they were manifest within and across cases over time in line with previous longitudinal IPA studies (e.g. Smith, 1994; Spiers et al., 2016).

An independent audit was carried out by a researcher not involved in the project. This involved examining both the process and narrative account in order to verify the trustworthiness and credibility of the findings. Smith (2011) notes the importance of providing some measure as to the prevalence of each theme within the data corpus and so, as suggested, extracts from at least half the participants are used to support each theme in the text.

## Results

The process of analysis produced two interrelated super-ordinate themes regarding older people’s experiences of oxygen concentrator use in the home. The interrelated super-ordinate themes were (1) the journey of acceptance and (2) negotiating changing relationships. The findings are presented as super-ordinate themes and sub-themes substantiated by extracts from participant interviews. Pseudonyms have been used to protect anonymity while u/p denotes user/partner. T1, T2, T3 indicates which interview the extract is taken from (see Table 1).

### The journey of acceptance

The longitudinal nature of this study captured the different steps or stages that participants had to negotiate on the journey to accepting the oxygen concentrator into their lives and homes. The different stages of this process are described in the sub-themes ‘initial reactions’ (relating to themes derived predominantly from the first interviews), ‘starting the journey’ (relating to extracts from both first and second interviews) and ‘along the path to acceptance’ (derived predominantly from the third interviews).

#### Initial reactions

The initial reactions of the participants were unsurprisingly related to the expectations they had about the device. Three of the couples seemed to have little idea about the device that was being given to them; in fact, they had only seen the concentrator for the first time during the assessment in the clinic. For those three couples, there was a real sense of shock that things had moved too quickly and they had in some way lost control over their situation.

For Ray and Rita, even though they themselves had been thinking about oxygen, the swiftness and reality of being given the device seemed to leave them in a state of shock, so much so that they were unable to ask anyone for information in a bid to better prepare themselves:

I never thought he’d get it … I were a bit shell-shocked, because I wasn’t expecting that at all … So, they said he needed it so I thought well we’d better get it in then … I were quite shocked when they said they were putting him on it. I never really got to ask the questions I might have asked. (Rita p T1)

This sense of shock was even more pronounced for Tracey who left her feeling scared. This sense of fear was disorientating in that she did not understand how she had got into this situation or indeed what, if anything, she could do next. It was as if she had lost some control over what was happening to her:

I felt frightened. There was something unknown to me … So [the concentrator] was just thrown on me … it frightened me to death … and I’m still frightened … at the moment I’m scared, I’m lost, I don’t know what I’m going to do next. (Tracy u T1)

The repeated use of the word ‘shocked’ by Rita and the phrase ‘just thrown on me’ by Tracy emphasises just how powerless they felt due to the sudden change in situation. There was a sense of being frozen and unable to see a way forward.

These feelings of shock and fear were manifested in the initial denial and rejection of the device for Wilma in couple 4. She associated the device with hospitals which brought home the seriousness of her condition. In fact, the device was installed while she was still in hospital with an exacerbation of her COPD and was a condition of her discharge home. The participant was particularly adamant about not needing the device or wanting it in her house that she became quite angry with medical staff:

I felt, no, I don’t need that! … I just have never seen anything like it for medical things … Things like that. That, to me, is like a hospital thing; should be in hospital if you’ve got something like that … I was absolutely gobsmacked when they told me. And I said to her I don’t want it, I said don’t bother bringing it because I don’t want it! She said well you have got to have it, I said I haven’t! I don’t have to have anything I don’t want. I said I don’t want it, I am not having that all over my house. (Wilma u T1)

Couple 2, who had some knowledge about the device and what it entailed, saw the situation in a much more positive light: the device for this user was something aspirational and longed for. Finally being given an oxygen concentrator was exciting and a great relief from the physical struggles of her current situation. Being given the device would provide the opportunity for greater freedom around the house:

I mean I had hoped before that that they would give me one actually, because I had heard about them before … And then when she told me that she was going to give me one, I thought yes, great! You know I am actually going to get somewhere now. (Sally u T2)

#### Starting the journey

Following the initial reactions, this part of the journey describes how the couples feel about having the device in their lives in the first few weeks following the delivery of the device. Ray and Rita in couple 1 differed considerably in their acceptance of the device in these early stages. Ray was particularly relaxed and seemed to take everything in his stride. This was reflected in the way he spoke about the device in the early interviews and how quickly the device just fitted in and was accepted as part of his life:

I mean you soon get used to that … So as regards the machine, there’s nothing, is there? … As I say, I can’t see any bad problems, not really … I’m used to it. It’s as if it’s been there for years. (Ray u T1)

This easy acceptance was not necessarily shared by Ray’s partner. The device had not become part of everyday life in the same way it had for her husband. In fact, she seemed to feel as if she has no choice but to accept the device. There is, however, the idea that the device would be more readily accepted if it could be seen to have some tangible benefits:

I still see it when I come in [to the room], I don’t think [he] sees it’s there … But I see it when I come in … Well, I’ve got to accept it … if I thought it was going to do him good, and we could go on holiday, then I’ll accept it … But no, at the moment, no, I don’t, no … To me I still look at it and think. I don’t think it shouldn’t be here, I just think. Oh dear. (Rita p T2)

Both Sally and Stan in couple 2 found accepting the device to be relatively easy. The reality of having the device in the home seemed to fulfil their expectations and support their initial reactions:

I wouldn’t say we’ve had to make any adjustments, have we, really? [It] Just fits in lovely, you know, what I need … I feel happy at being on it … just gives me peace of mind. (Sally u T2)

While Stan did not receive any direct benefit from having the device with which to help him accept the device, he was particularly pragmatic when it came to the device and his wife’s needs:

I get on with it alright. It’s something she’s got to have and it helps her, that’s it. She needs it, so if you need it you have to use it … she needs it, so that’s how I just accept it. (Stan p T1)

Couple 4 spoke about their struggle in accepting the device. Having the concentrator in their lives and a general worsening of her condition seemed to signify a transition from the past into a new way of living:

I don’t know. I really had no idea about it, none at all. I know at the hospital it comes out of the wall … but at home I never envisaged – honestly, I couldn’t even contemplate anything like that … It’s just – it’s like a whole new world, isn’t it? (Wilma u T1)

It’s like I say, I didn’t visualise [her] having this on all the time. (Wally p T1)

For the user, the device acted as a reminder of her relatively recent active life and marked the transition from a life of active independence to one of dependent inactivity. For her, accepting the device seemed to entail a long psychological struggle of coming to terms with this new life:

I honestly and truly think a lot of it is psychological as well because it reminds you all the time of what you can’t do, not what you can do, but what you can’t do … But now I can’t really do anything. (Wilma u T1)

The fact that neither of them had explained the situation fully to their families signifies a certain amount of reticence and the clinging on to the idea that things might revert back in the future so there is no reason to worry other family members needlessly:

I don’t think I’ve really spoken to [my family] and said, ‘[Wilma] has got to have this on for the rest of her life’. I don’t know. I don’t know whether I would like to say that just yet just in case something changes. (Wally p T2)

Yeah. I haven’t said it to mine that it’s most probably a permanent … I just want to accept it, I suppose, before anything else … It’s a big thing really, isn’t it? I think it is. If I can’t really accept it yet you can’t expect them to. Just have to wait and see. (Wilma u T2)

#### Along the path to acceptance

For Sally and Stan, the journey of acceptance had been relatively easy and the final interview reports how the device has been normalised and exists in the background of their lives:

I mean I’ve got used to it, it’s become part of the norm now and I can’t do without it … As I say it just all sort of fits into your life and it just becomes normal. (Sally u T3)

[The concentrator is] not too noisy, get use to that and that just becomes background noise after a while, you don’t even hear it. (Stan p T3)

However, the initial excitement of finally being given an oxygen concentrator and the comparative ease of transition into their lives may have masked some of the frustrations associated with the device. It seems that once the novelty had worn off, some irritations became harder to ignore:

but it is starting to frustrate me because of this [tubing]everywhere, I get it stuck under doors and wrapped round things, and then I could be walking across the floor and all of a sudden dogs will stamp on it and I’m stuck … now it’s like become part of the norm and you start picking on things. (Sally u T3)

Ray and Rita’s final interview was conducted earlier than expected as following a further medical assessment, the device had been removed from home. This, however, provided a valuable insight into how accepted the device had become.

The device seemed to have become quite ingrained and had become part of the routine of the user’s life. Even after the device had been removed, he still looked to use his oxygen in the mornings:

The first two days he kept saying, ‘I’ll go and put my gas on’. (Laughter) [He] kept trying to put his gas on. (Rita p T3)

This feeling wasn’t shared by the partner. As in the previous interview, she still hadn’t accepted the device fully. This was not necessarily down to a conscious resistance; in fact, she fully expected it to become part of their lives and seemed disappointed that it had not; however, she needed more time for this process to become complete:

As I say, we didn’t have it long enough for it to become part of us … I know people who have had them for years and it’s part of your life, isn’t it? I thought that were going to be part of our life … And it hasn’t, it hasn’t worked out. (Rita p T3)

For both participants, the removal of the device seemed to be quite a dramatic event:

Rita (p T3): So when we went again and they reassessed it, they decided that, well to take it off him … And they came in in two days and took it.

Ray (u T3): At the end of the day it’s what they want to do … They snatch it back.

Rita (p T3): Then we’ve got to just [manage].

Instead of being happy that Ray no longer needed to use the device any more, the idea that the concentrator was ‘snatched’ away implies that it was somehow stolen from them and they were now being left to cope on their own. This not only suggests a sense of ownership but that the device was in fact valued by them both.

Similarly, couple 4, who seemed to have the most difficult transition towards accepting the device into their lives, seemed to have adopted it as part of the family:

The thing is, with the machine I don’t even notice it’s there now, it’s just part of the family. (Wally p T3)

It’s like having a dog! … You don’t really notice it that much now though, do you? (Wilma u T3)

The likening of the medical device to a pet is interesting as it implies a kind of domestication of ‘the medical’ into ‘the familial’. So instead of a big, grey medical device being conspicuous within the home, the device is seen as being belonging and being part of its surroundings.

The device had become so much part of the background of their lives that ironically now it was the absence of the device noise that signalled its presence:

No, it’s funny, it’s just there. When it goes off you kind of think, ‘Oh, isn’t it quiet?’ It’s really weird, isn’t it? … Whereas before, when we first had, it we used to think ‘Isn’t it noisy’. So you do – your mind changes, actually … It’s really weird. (Wilma u T3)

This super-ordinate theme has described a journey or transition that the participants undertook as they come to accept the device into their lives. The following theme describes the changes in couples’ relationships that had to be negotiated as they learned to accept life with an oxygen concentrator.

### Negotiating changing relationships

Accepting the oxygen concentrator into their lives involved the participants having to negotiate changes in their relationships with the people around them. The device was found to be both a source of conflict and of harmony and also added to the emotional burden endured by the partners.

#### A source of conflict and harmony

On the whole, for couple 1, the device was found to help reduce tensions between the participants. A result of using the oxygen concentrator was that the user was more awake during the day which had been a particular area of tension between the couple:

Int: So with him being awake a bit more during the day how did that impact on your lives?

Rita (p T3): Well lovely because I don’t get so frustrated … It [was] very frustrating. I [would] sit for hours, you know, and you get no sense out of him, and I’ll have a look round and he’s gone [to sleep] again … [With the concentrator] I didn’t get so frantic. I admit, I used to get frustrated then I get nasty with him.

For couple 2, there were aspects of the device that caused a certain amount of conflict. Maintaining the device entails changing a little filter at the back of the concentrator, a task that the partner did every week. While this is not a particularly big job, it was obviously a contentious issue:

Stan (p T2): It is because I know for a fact she won’t change it!

Sally (u T2): I would, if I thought about it!

Stan (p T2): She would wait until it got real solid with dust.

Sally (u T2): No, you are making out I am a dirty minger!

Stan (p T2): She will say there is something wrong with my oxygen, I am not getting enough. And it will be the filter!

Another area of device use that caused arguments was around the dangers of using the oxygen in the kitchen:

Sally (u T2): … a couple of times I have mistakenly gone in there and think oh no I have got my mask on! Take it off …

Stan (u T2): She has had enough warnings and bollockings about going in there with it.

Sally (u T2): And sometimes I just walk in and forget!

Stan (u T2): You walk in one day with that on, the gas is on, I said there will be an almighty bang and I shall be kissing your arse goodbye. There will be nothing left of the house.

Stan seems particularly irritated with what he perceives as Sally’s lack of concern surrounding this hazard. While she mentions a couple of instances of forgetting, his issuing of ‘enough warnings’ and graphically pointing out the direct consequences of her actions demonstrates how Stan seems to have taken responsibility for supervising this facet of concentrator use.

Couple 4’s area of tension was related to the partner’s extra responsibilities associated with helping with the device and extra jobs around the house:

Wally (p T2): We do get a bit trite with one another, now and again.

Wilma (u T2): Tetchy.

Wally (p T2): What were you shouting to me this morning? [the piping] has got jammed, then I have got to [sort that out]

Wilma (u T2): You thought you had got rid or her for a few minutes, having a shower.

Wally (p T2): I do get a bit uptight sometimes, and I think oh I am just going to do the washing up, and I am going to do this, I am going to do that.

While Wally has other chores around the house, he is needed to attend to the piping when it gets stuck as Wilma moves around the home, and this demonstrates how Wally is himself also connected to the concentrator. It seems as if his own freedom is curtailed by the device because Wilma’s freedom to roam is reliant on him being available to free the piping. It is unclear whether such innocuous terms such as ‘bit trite’, ‘tetchy’ and ‘uptight’ fully represent how they feel or were used to down play the situation due to interviewing both patient and partner together.

Couple 3 was particularly concerned about the impact the concentrator would have on their relationship with their son. There had been an issue of the son smoking in the house previously, but with the danger of smoking around the oxygen concentrator, they were worried that it may discourage the son from visiting and leave them even more socially isolated:

Terry (p T1): No, we don’t have any visitors.

Tracy (u T1): I don’t see anyone.

Int: What did your son did you say about the concentrator?

Tracy (u Y1): The only thing he’ll turn round and say is, ‘well I’m not coming down any more if I can’t smoke’.

Terry (p T1): So he says he shan’t come any more. So that’s it then.

However, the reality was that the introduction of the concentrator seemed to act as a prompt for the son to start to stop smoking:

Tracy (u T2): [After seeing the device] I think he knew very well he had got to pack his cigarettes up! I think that is all he thought of.

Terry (u T2): In the past, when we have said something about smoking and we told him not to smoke in here, he said if I can’t have a fag, I am not going to come anymore, this sort of thing. But it is possible that through this [the concentrator] …

Tracy (u T2): … because he has not been able to smoke whilst he has been down here, you see.

Terry (u T2): We think he has finally got round to doing something [about stopping smoking].

#### Increasing emotional burden

The oxygen concentrator provokes an increase in the emotional burden experienced by the partners of the users. For couples 1 and 2, the device seemed to put a lot of focus on the user, the result of which meant that the partners received less attention and in some way felt forgotten about. The partners each had their own life events which had become marginalised since the device had entered their lives. The partner in couple 1 reported a fall that she had suffered but felt regardless of her own condition, her husband’s needs were more important:

I fell three weeks ago. I tripped on the stair … and there then I’m thinking, ‘I can’t go to hospital’. I sat on the [step] and I thought, ‘I can’t because who’s going to look after him?’ … ‘But it’s not about me, it’s about him. Life’s about [him]’. (Rita p T3)

Similarly, the partner in couple 2 had his own health problems. While the interviews were obviously centred on the device, the partner would talk at length about his own health problems whenever the opportunity arose. He would go into great detail, as if it was a way for him to be able to express the importance of his own illness which had no visual cues (such as a medical device) to its severity:

Well, I came out of hospital, had my operation, had problems on recovery, had a bleed into the heart which wouldn’t stop for a minute … I was on a different pain killer, which is class A and I had another class A I picked up from the chemist which I shouldn’t have done … (Stan p T3)

In fact, he was adamant that his own health should be considered equally important:

Stan (p T2): We are just hoping her appointment doesn’t come when mine does, aren’t we?

Sally (u T2): Because he is waiting for an operation as well, so we are hoping they don’t clash!

Stan (p T2): I am not cancelling it.

The emotional burden for the partner in couple 4 was quite different. First, the concentrator acts as a reminder to Wally of the speed of his wife’s decline in health, and the associated low moods were something that, due to their social isolation, he alone is witness to. It is he who is required to provide the support she needs to get through these bleak periods:

I certainly do worry about it as well because seeing [her] go down so quick it does worry you … Sometimes really she has her dark times when nobody is here, nobody else sees the bad times apart from me. [You] just got to fight on. (Wally p T1)

Adding to his worries about Wilma was the fact that using the device could be dangerous and that seemed to make him more sensitive to quite normal situations:

It worries you sometimes, because they do say that [with the concentrator] if you fall asleep, or in the daytime or anything else, the first sign is the carbon dioxide building up, making you drowsy, and when she oversleeps sometimes, I get a bit worried, because I think maybe something is not right. (Wally p T2)

## Discussion

The aim of this study was to describe the experience of being provided with an oxygen concentrator to use in the home from the perspective of both the user and their partner.

The super-ordinate theme ‘the journey of acceptance’ described how the couples moved through the process of accepting the device into their lives. Acceptance has often been discussed in relation to illness and disability and has been identified as an important factor in relation to psychosocial adaptation in chronic disease (Wright and Kirby, 1999).

The ease with which the device was accepted by these couples appeared to be mediated by the expectations that they had about the device. Indeed, the three couples who did not know what to expect when receiving the oxygen concentrator engaged in more dysfunctional psychological strategies such as rejection. This was particularly well illustrated by Wilma who did not even want the concentrator in the house. These couples had to negotiate their way through the process of accepting this device. Wilma and Wally demonstrate some resolution to their dilemma when the device is likened to a family pet. Sally and Stan, who had a better idea of what to expect through informal or self-education, initially experienced an easier acceptance process. However, as time went on, certain aspects of living with the concentrator seemed to become more irritating. This could be an indication of having unrealistic expectations surrounding the device.

Patient expectations are seen as exerting an important influence on healthcare experience and satisfaction and that information given to patients can help manage and modify those expectations (Conway and Willcocks, 1997; Oterhals et al., 2006) and play an important role in the acceptance process (LaChapelle et al., 2008).

Patient education in relation to LTOT in the United Kingdom is mainly concerned with practicalities, safety and encouraging treatment adherence (British Thoracic Society, 2006). While this content and level of patient education is undoubtedly important, it seems that there is little done to prepare couples for the psychological consequences associated with initiation of LTOT and the installation of the oxygen concentrator.

In order to facilitate acceptance and inform expectations of LTOT, the education of users and partners should occur prior to delivery of the device. The practicalities of this may be problematic under the current system. As per the guidelines, patients are assessed twice approximately 4 weeks apart in order to ascertain suitability for oxygen therapy. If after the second assessment LTOT is indicated, the equipment is ordered and is delivered to the patient’s home within 3 working days. This gives little time for either the delivery of a comprehensive education package or time for the patient to process the information given to them and make informed decisions about whether to accept the treatment or make preparatory adjustments to the home.

Participants in this study were given practical information regarding the device at the final assessment clinic appointment (prolonging life, not smoking, cleaning instructions, etc.), and again upon installation of the concentrator in the home, the emphasis was, not surprisingly, on the positives with little if any mention of any negatives.

Chronic disease not only has direct consequences for the chronically ill person but can also have a dramatic effect on the lives of people who care for them, which in many cases are their partner or spouse. The theme ‘negotiating changing relationships’ describes the different ways in which the device impacted on couples’ relationships with each other. A number of qualitative research studies have provided descriptions of the relationship between those who care for and patients with COPD. These studies have described the increasing physical and emotional burden placed on partners caring for people with COPD and the tensions that can arise within those relationships. For example, Rita talking about how her own physical health being of secondary importance to that of her husband is similar to the findings of Bergs (2002). Gabriel et al. (2014), Kanervisto et al. (2007) and Seamark et al. (2004) describe the role dysfunctional communication has on increasing tensions between patients and carers living with COPD. The present study not only adds to this picture but also goes on to describe novel findings about the ways in which the oxygen concentrator itself can contribute to or alleviate some of these issues. For Stan and Sally, arguments about who is responsible for cleaning and maintaining concentrator and also the very real danger of using the device in the kitchen show how the device can be the cause of disharmony. For Rita, however, the concentrator changed the way she behaved towards her husband. She admitted to being frustrated and ‘nasty’ with him before the device and that her relationship with him had improved.

The findings of this study support those of Gale et al. (2015) whose study describes the process of COPD patients’ adaptation to non-invasive ventilation and calls for changes in education and practical support to better facilitate adaptation to that particular therapy. The implications in relation to LTOT are that patient and partner education that not only focuses on the practicalities and positives of device use but also adequately addresses psychological and social issues associated with using oxygen concentrators may better prepare couples to manage their expectations and facilitate accepting the device into their lives.

Since this piece of qualitative research has a small sample size, recommendations should be viewed with caution and seen as a starting point leading to further research. For example, it would be beneficial to follow this up with a larger study specifically designed to compare and contrast different content and modes of delivery of patient education and how they impact on couples’ acceptance of oxygen concentrators.

## Study limitations

Due to the short time between patients’ final assessment and the device being delivered into the home, we were unable to interview all couples before the device was delivered. Also, two of the couples had the device removed before the end of the study, which impaired some of the longitudinal aspects of the study. However, in the case of couple 1, this proved to be an unplanned advantage as it offered the opportunity to discover their reaction to this event and what the device had come to mean to them.

The device user and their partner were interviewed together for this study. This did facilitate some good discussions and allowed the researcher to see how they reacted to each other, but it may have resulted in a less open discussion. There was, for instance, no mention of how the device may have impacted on their feelings towards each other or in relation to intimacy. It may also have made it more difficult for the partner to express openly negative feelings or emotions about the device which the user was reliant on for fear of upsetting that person or making them feel guilty.

## Conclusion

The study has described the complexities experienced by couples where one person is prescribed an oxygen concentrator for LTOT. It seems that managing patient and partner expectations of life with an oxygen concentrator may ease the process of accepting their new situation. The education that may be most beneficial should not only concern the practicalities but should also cover the psychological and social impact that the device may have. Future studies that consider different educational content and how and when that content is delivered may prove useful in facilitating the acceptance process.
